# Comparing patient care seeking pathways in three models of hospital and TB programme collaboration in China

**DOI:** 10.1186/1471-2334-13-93

**Published:** 2013-02-20

**Authors:** Xiaolin Wei, Guanyang Zou, Jia Yin, John Walley, Qiang Sun

**Affiliations:** 1 School of Public Health and Primary Care, The Chinese University of Hong Kong, 2/F, School of Public Health and Primary Care, Prince of Wales Hospital, Shatin, Hong Kong, N.T, China; 2 Nuffield Centre for International Health and Development, University of Leeds, 101 Clarendon Rd, LS2 9LJ, Leeds, UK; 3 Center for Health Management and Policy, Shandong University, No 44 Wenhua Rd, Mailbox 128, Jinan, Shandong 250012, China

**Keywords:** Tuberculosis, Care pathway, Hospital TB collaboration, China

## Abstract

**Background:**

Public hospitals in China play an important role in tuberculosis (TB) control. Three models of hospital and TB control exist in China. The dispensary model is the most common one in which a TB dispensary provides both clinical and public health care. The specialist model is similar to the former except that a specialist TB hospital is located in the same area. The specialist hospital should treat only complicated TB cases but it also treats simple cases in practice. The integrated model is a new development to integrate TB service in public hospitals. Patients were diagnosed, treated and followed up in this public hospital in this model while the TB dispensary provides public health service as case reporting and mass education. This study aims to compare patient care seeking pathways under the three models, and to provide policy recommendation for the TB control system reform in China.

**Methods:**

Six sites, two in each model, were selected across four provinces, with 293 newly treated uncomplicated TB patients being randomly selected.

**Results:**

The majority (68%) of TB patients were diagnosed in hospitals. Patients in the integrated model presented the simplest care seeking pathways, with the least number of providers visited (2.2), shortest treatment delays (2 days) and the least medical expenditure (2729RMB/401USD). On the contrary, patients in the specialist model had the highest number of provider visits (4), longest treatment delays (23 days) and the highest medical expenditure (11626RMB/1710USD). Logistic regression suggested that patients who were hospitalised tended to have longer treatment delays and higher medical expenditure.

**Conclusion:**

Specialist hospital treating uncomplicated cases not using the standard regimens posed a threat to TB control. The integrated model has shortened patient treatment pathways, and reduced patient costs; therefore, it could be considered as the direction for future reform of China’s TB control system.

## Background

Tuberculosis (TB) is a global public health threat, with 8.7 million TB patients and 1.4 million deaths due to TB estimated in 2011. Over 95% of TB deaths occurred in low and middle income countries [1]. Global TB control adopts a systematic care approach called DOTS (Directly Observed Therapy, Short-Course) programme, which has been recommended by the World Health Organisation (WHO) since the 1990s. The DOTS programme has been largely implemented by public sectors under national TB programmes (NTPs). However, many patients seek care from a variety of health providers, often outside of the NTP’s TB clinics. These providers include public hospitals, corporate health services, private clinics and hospitals [2]. In many developing countries, the public-private/public mix (PPM) approach were promoted to extend effective standard TB care from the NTP clinics to all other providers [3]. Studies have demonstrated the PPM strategy has contributed to detecting more TB patients, reaching a wider population, and achieving better treatment outcomes [4-6], as well as being cost effective at the same time [7-9].

In China, public hospital is the largest healthcare provider, but it is often not part of the NTP. China’s NTP is organised in a semi-vertical system by TB dispensaries at four levels: national, provincial, prefecture and county/district. The majority of patients are diagnosed and treated at the district/county TB dispensary, usually a department located in the local Centre for Disease Control. TB dispensaries at higher levels mostly provide programme administration, case reporting and service supervision. Free TB DOTS service is provided in TB dispensaries, covering costs of the first-line anti-TB drugs, two X-ray examinations and all sputum smear examinations [10]. Patients who seek care outside of TB dispensaries are not covered by the free treatment policy. Historically, there was a lack of coordination between public hospitals and the NTP in China, resulting in patients’ long diagnostic delays, poor treatment outcomes and high patient financial burdens [11]. The 2000 national TB survey reported that 91% of TB patients visited public hospitals first to treat TB related symptoms, while only 25% of TB suspects in public hospitals were referred to TB dispensaries [12]. Recent studies reported that over 70% of TB patients in public hospitals were not referred to TB dispensaries [13], while many patients spent over half of their annual income in public hospitals before their TB diagnoses [14-16].

The government has promoted hospital and TB collaboration since 2006 [17]. Under the WHO PPM framework, three models of hospitals and TB collaboration in China are identified. The dispensary model is the most common one in which TB dispensaries provide all TB care regarding diagnosis, treatment, clinical management, as well as health education, supervision and case reporting. Public hospitals are responsible to refer TB cases to TB dispensaries, and TB dispensaries should trace all referred cases who have not visited the dispensary within three days. Only complicated and severe TB cases should be treated in general hospitals. The specialist model is similar to the former except that a specialist TB hospital is located in the same area. The specialist hospital should only treat severe TB cases according to national guidelines and refer uncomplicated patients to the TB dispensary [18]. This model exists mostly in north China and some provincial capitals. The integrated model is a new initiative to integrate TB care within public hospitals. A clinic is set up in a general hospital to provide TB clinical care. The hospital becomes known as a TB designated hospital. TB patients are diagnosed, treated and followed-up in the designated hospital. In this model, the TB dispensary is responsible for TB public health care, including training, education, case supervision, cohort analysis and reporting. Other public hospitals should refer TB suspects and patients to the designated hospital. This model has been practiced in Shanghai, Zhejiang and Jiangsu provinces, and a few sites in less developed western provinces.

Current policy debates were regarding which model fits into China’s future TB control, because the TB dispensary is not prepared for the emerging of multi-drug resistance (MDR) TB, TB/HIV and other complicated cases. Furthermore, the dispensary model was at stake after the enactment of the Law for Licensing Medical Practitioners which banning public health doctors in TB dispensaries providing clinical care [19]. The study aims to analyse and compare the care-seeking pathways and medical expenditureof TB patients under the three hospital TB collaboration models in China, and to provide policy recommendations for the future of TB system reform in China.

## Methods

### Setting

A cross-sectional survey was conducted in the six sites of China. Two sites of each model were suggested by the national and provincial TB programmes, with one relatively rich, and the other relatively poor. Two sites of the integrated sites were selected in Shanghai (east) and Guangxi (west) because the integrated model was new in China and both sites had been established by local health authorities over three years. Two of the specialist sites were selected in Shandong province, as this model was most popular in northern China. For the dispensary model, one site in Zhejiang (east) and the other in Guangxi (west) were selected. Each site consists of a county or a district where the basic TB management unit is located. National TB guidelines have been implemented across all China; therefore, standard TB diagnosis and treatment should be provided in all six sites [20].

### Study participants

Due to the availability of TB cases, we randomly selected 50 TB patients in each site from the TB dispensary registers according to the inclusion criteria: 1) being new sputum smear positive or negative pulmonary TB patients, 2) been registered in 2007 and successfully completed treatment by August 2008; 3) having no records on any serious co-morbidities such as diabetes, cardiovascular disease, hepatic disease or severe respiratory symptoms. All selected cases treated by the specialist hospitals were negative in drug sensitivity tests. Drug sensitivity tests were not available to TB patients in other models. But we selected cases which had been successfully treated, so they were not likely to be drug resistant.

### Data collection

Subjects were surveyed using a structured questionnaire adapted from the National TB patient survey [12], which had been used in other similar settings [14,21]. Questions covered patient social-economic status, experience of TB diagnosis and related treatment, patient expenditure on TB related treatment, i.e., excluding any coverage from insurances, and delays. Patients were selected by a random number from the TB registers, and then were invited to the place where they were treated before, i.e., the TB dispensary, the specialist hospital or the TB designated hospital. A free consultation was provided as an incentive. Taking consideration that 10% of patients may not come to the survey, each site invited 55 patients. In total, 293 patients were surveyed, including 100 in the dispensary sites (51 from the rich site and 49 from the poor site), 90 in the specialist sites (44 and 46, respectively) and 103 in the integrated sites (50 and 53, respectively).

Ethical approval was granted by the Ethical Committee for Health Policy Studies located in Shandong University. Written informed consents have been obtained from all participants in the study. Data were collected by a team of experienced researchers and postgraduate students in 2008.

#### Analysis

SPSS 14.0 (Chicago, USA) was used for analysis. Chi-square test, Kruskal-Wallis H test and ordinal data analysis were employed when appropriate. Logistic regression was employed to identify factors related with treatment delay (0 = ≤7 days; 1 = >7 days) and medical expenditure (0 = ≤3000 RMB/ 441 USD; 1 = >3000 RMB). Independent variables were selected based on individual regression with the dependant variables at the significant level of α = 0.1 and their theoretical relevance. Dummy variables were generated for variables having more than 2 values. The two logistic models were statistically significant (model coefficient tests P < 0.05) and showed a good model fitness (Hosmer and Lameshow tests, P > 0.90).

We defined the place of actual TB diagnosis as where the patient was first informed of TB with sputum microscopy examined. Treatment delay refers to the period after TB diagnosis and before the standard DOTS chemotherapy initiated in TB dispensaries or designated hospitals. Treatment delay is considered long if over one week [13]. We also examined diagnosis delay, which is defined as the period from the first healthcare contact until the patient’s TB diagnosis. We used 3000RMB/441USD as the cut-off point of medical expenditure in the logistic regression as 3000RMB is the median medical expenditure of all patients. Health providers were grouped as primary care facilities (village doctors, township hospitals and community health centres), hospitals, specialist hospitals, TB dispensaries or TB designated hospitals. Repeated visits to the same health provider, e.g., renewing drugs, were accumulated and regarded as one visit in the pathway map; however, different diagnoses with the same provider were accounted as different visits to highlight a possible delay during the care.

## Results

Of the 293 patients participated in the study, the average age were 47 years old. Per capita income was the highest in the integrated model. The majority of patients in the dispensary and specialist sites were farmers. Most patients were covered by insurance such as the basic health insurance in urban areas and the new cooperative medical insurance scheme in rural areas (Table 1).

**Table 1:** General information of patients participated in the survey under the three models

	**The dispensary model**	**The specialist model**	**The integrated model**
Patients survey	100	90	103
Average age $\bar{\text{x}}$ (95%CI)	53 (49.6-56.1)^a^	46 (41.8-49.8)	42 (38.8-45.1)
Male, N (%)	62 (62)	62 (69)	65 (63)
Married, N (%)	79 (79)	58 (64)	72 (70)
Farmer, N (%)	67 (67)	51 (57)	24 (23)^b^
With medical insurance, N (%)	94 (94)	82 (91)	77 (75)
Per capita annual income (RMB) $\bar{\text{x}}$ (95%CI)	5,146 (3481-6811)	4,226 (2930-5523)	11,197 (8516-13878)^c^

1 USD = 6.8 RMB.

^a^ Kruskal-Wallis H test showed that significant difference was found among three models (χ^2^ = 36.448, P<0.001). The average age of patients in the dispensary model was significantly higher than that in the specialist model (P = 0.003) and the integrated model (P<0.001).

^b^ Significant lower than the dispensary model (χ^2^ = 39.176, P<0.001) and the specialist model (χ^2^ = 22.506, P<0.001).

^c^ Kruskal-Wallis H test showed that significant difference was found among three models (χ^2^ = 21.056, P<0.001). Per capita income of patients in the integrated model was significantly higher than that in the dispensary model (P<0.001) and the specialist model (P<0.001).

### Patient pathways until treatment completion

On average, a patient in the specialist model visited four health providers until treatment completion compared with only 2.2 providers for a patient in the integrated model (P < 0.01). Over 70% of patients in the dispensary and integrated models visited one provider until diagnosis of TB (Table 2). The majority (68%) of TB patients were diagnosed in public hospitals: 37% of patients in the dispensary model were diagnosed in general hospitals, 56% in the specialist model were diagnosed in specialist hospitals, and 93% in the integrated model were diagnosed in the designated hospitals.

**Table 2:** Number of health providers visited from first contact of care until treatment completion by TB patients in the three models

	**The dispensary model**	**The specialist model**	**The integrated model**
Patient survey	100	90	103
Total health providers per patient visited	2.6	4.0^a^	2.2
1-2 times (%)	51 (51)	16 (18)	75 (73)
>2 times (%)	49 (49)	74 (82) ^b^	28 (27)
Health providers per patient visited before diagnosis	1.1	1.8	1.0
1 times (%)	70 (70)	43 (48)	82 (80)
>1 times (%)	30 (30)	47 (52)^c^	23 (22)

^a^ Kruskal-Wallis H test showed that significant difference was found among three models (χ^2^ = 85.962, P<0.001). Patients in the specialist model visited more health providers than patients in the dispensary model (P<0.001), and so was the dispensary model compared with the integrated model (P = 0.005).

^b^ Proportion of over 2 times in the specialist model was significantly higher than that in the dispensary model (χ2 = 22.902, P<0.001), and the proportion in the dispensary model was significantly higher than that in the integrated model (χ2 = 10.257, P = 0.001).

^c^ The proportion in the specialist model was significantly higher than that in the dispensary model (χ2 = 9.705, P = 0.002) and the integrated model (χ2 = 19.357, P<0.001).

**Figure 1:**
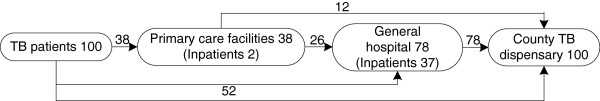
Care pathways of 100 tuberculosis (TB) patients in the dispensary model from first contact care until treatment completion.

In the dispensary model (Figure 1), 52% (52) of patients used general hospitals as their first contact of care, because of the perceived high quality (75%, 39/52) of the general hospitals. Thirty-eight percent of patients firstly visited primary care facilities such as township hospitals or village doctors, while 10% firstly visited the county TB dispensary. All the 78 patients who visited the general hospital were referred to TB dispensaries, but 47% of them were hospitalised before the referral.

**Figure 2:**
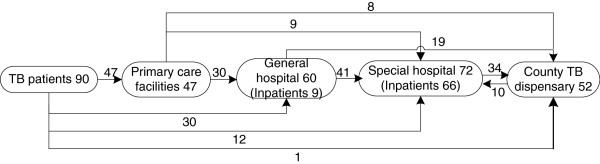
Care pathways of 90 tuberculosis (TB) patients in the specialist model from first contact of care until treatment completion.

In the specialist model (Figure 2), 52% (47) of patients firstly visited primary care facilities for convenience reasons (77%, 36/47). Thirty-three percent firstly visited general hospitals, while 13% firstly visited the specialist hospital. Of the 60 patients who visited general hospitals, 68% continued to visit specialist hospitals. The majority (92%, 66/72) who visited specialist hospitals were hospitalised there. Only 47% (34/72) were referred to the TB dispensaries. Ten patients visiting the TB dispensaries continued seeking treatment in the specialist hospitals due to the latter’s high fame in TB care.

**Figure 3:**
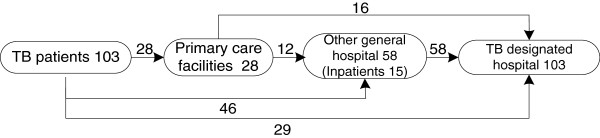
Care pathways of 103 tuberculosis (TB) patients in the integrated model from first contact of care until treatment completion.

In the integrated model (Figure 3), 28% (29/103) firstly visited the TB designated hospitals, while 45% (46/103) of patients started their first treatment in other general hospitals. Reasons of visiting the TB designated hospital included perceived high quality of care and closeness to patient’s homes. All the 58 patients who visited other general hospitals were referred to the TB designated hospitals, but 25% were hospitalised in these hospitals before being referred to the designated hospital.

Table 3 showed that patients who visited hospitals had higher medical expenditure than those who did not, as shown in the dispensary and specialist models (patients pathways 1, 2 vs. patients pathways 3,4,5,6, P < 0.05). In the specialist model, patients who only received care in the specialist hospital spent much more expenses than those who received DOTS treatment in TB dispensaries (patients pathways 5 and 6 vs. other patients pathways, P < 0.05). Patients who visited primary care facilities had longer diagnostic delay than patients who did not in all the three models (P < 0.001). Average medical expenditure were the highest in the specialist model (RMB 11626/USD 1710, P < 0.01) and the lowest in the integrated model (RMB 2729/USD 401, P < 0.01).The delays were significantly shorter in the integrated model compared with that in the dispensary model and the specialist model (P < 0.05).

**Table 3:** Patient major care pathways, their medical expenditure and delays in the three models of hospital and TB linkages in China

**Pathway**	**The dispensary model**				**The specialist model**				**The integrated model**			
	**Cases, N (%)**	**Average medical expenditure**$\bar{x}$**(RMB)**	**Days of diagnosis delay**$\bar{x}$**(M)**	**Days of treatment delay**$\bar{x}$**(M)**	**Cases, N (%)**	**Average medical expenditure**$\bar{x}$**(RMB)**	**Days of diagnosis delay**$\bar{x}$**(M)**	**Days of treatment delay**$\bar{x}$**(M)**	**Cases, N (%)**	**Average medical expenditure**$\bar{x}$**(RMB)**	**Days of diagnosis delay**$\bar{x}$**(M)**	**Days of treatment delay**$\bar{x}$**(M)**
1.CTD/TDH	10 (10)	3663	11 (2)	5 (1)	1 (1)	2700	11	4	29 (28)	2296	3 (1)	2 (1)
2.PCF → CTD/TDH	12 (12)	3036	102 (30)	6 (1)	6 (7)	2353	25 (16)	0 (0)	16 (16)	2189	6 (2)	1 (1)
3.Hospital → CTD/TDH	52 (52)	5055	15 (2)	9 (1)	22 (24)	10029	8 (1)	20 (15)	46 (45)	2512	12 (2)	3 (1)
4.PCF → Hospital → CTD/TDH	26 (26)	4972	53 (10)	25 (8)	23 (26)	11373	52 (20)	32 (8)	12 (12)	5327	33 (25)	3 (1)
5.Specilaist Hospital*	0				20(22)	9547	2 (1)	-	0			
6.PCF → Specialist Hospital*	0				18 (20)	19798	29 (9)	-	0			
Total	100	4652	35 (4)	12 (1)	90	11626^a^	23 (6)	23 (0)	103	2729	11 (1)^b^	2 (1)^c^

PCF: Primary care facilities, including village doctor clinics and community health centres or township hospitals.

CTD: county TB dispensaries; TDH: TB designated hospitals.

Hospital: here including general hospitals and TB special hospital, but excluding TB designated hospitals.

* For patients finished their treatment in the specialist hospitals, including pathways from general hospital to the TB special hospital and from primary care facilities to general hospital, then to the TB special hospital.

^a^ Kruskal-Wallis H test showed that significant difference was found among three models (χ^2^ = 83.268, P<0.001). The specialist model was significantly higher than the dispensary model (P<0.001), and the dispensary model was significantly higher than the integrated model (P = 0.034).

^b^ Kruskal-Wallis H test showed that significant difference was found among three models (χ^2^ = 17.042, P<0.001).. The integrated model was significantly shorter than the dispensary model (P = 0.001) and the specialist model (P = 0.002).

^c^ Kruskal-Wallis H test showed that significant difference was found among three models (χ^2^ = 9.283, P<0.001). The integrated model was significantly shorter than the dispensary model (P = 0.024) and the specialist model (P = 0.005).

### Factors related with treatment delay and medical expenditure

Table 4 listed all the variables included in the logistic regressions, while Table 5 showed the significant variables included in the logistic regression models and their odds ratios. After adjusting demographic and socio-economic factors, patients in the specialist model were more likely to have treatment delays longer than one week and had higher medical expenditure. Patients who were hospitalised tended to have treatment delays longer than one week and higher medical expenditure. Patients who visited general hospitals or the TB specialist hospitals tended to have longer treatment delays.

**Table 4:** Dependent and independent values for logistic regression models

	**Indicators**	**Category**
values	Treatment delay	0 = ≤ 1 week; 1 = > 1 week
	Medical expenditure	0 = ≤ 3000 RMB; 1 = > 3000 RMB
Independent variables	Gender:	0 = female; 1 = male
	Age group:	0 = ≤24 years; 1 = 25-59 years; 2 = ≥60 years
	Marital status	0 = single; 1 = married
	Profession:	0 = farmer; 1 = else
	Annual per capita income:	0 = the lower 50% income group; 1 = the higher 50 % income
	If having medical insurance:	0 = no; 1 = yes
	Hospitalisation:	0 = no; 1 = yes
	Number of health providers visited:	0 = ≤2 times; 1 = >2 times
	If first contact as a primary care facility:	0 = no; 1 = yes
	If visited a general hospital or specialist hospital:	0 = no; 1 = yes
	Group:	0 = the integrated model; 1 = the dispensary model; 2 = the specialist model
	Diagnostic delays*:	0 = ≤ 2 weeks; 1 = > 2 weeks
	Treatment delays*:	0 = ≤ 1 week; 1 = > 1 week

* Only in the regression model of medical expenditure.

**Table 5:** Significant independent variables in the two logistic regression models

**Dependent variables**	**Independent variables in the equation**	**Odds ratio (95% CI)**	**P value**
Treatment delay	Group (comparing with the integrated model)		0.094
	- The specialist model	4.25 (1.12-16.17)	0.034
	- The dispensary model	3.41 (.98-11.78)	0.053
	Hospitalization	9.74 (4.15-22.83)	<0.001
	Visited the general or TB special hospital	5.23 (1.06-25.91)	0.043
Medical expenditure	Group (comparing with the integrated model)		0.026
	- The specialist model	2.75 (0.97-8.17)	0.038
	Number of health providers visited	5.38 (2.58-11.23)	<0.001
	Hospitalization	21.52 (6.04-76.70)	<0.001
	Treatment delay	5.42 (1.72-17.06)	0.004

## Discussion

Evidence from the study suggested that patients of the integrated model had the simplest care pathways, reflected by the least number of providers visited and the least medical expenditure spent. Early symptoms of TB are non-specific, including cough, fever and weigh loss. They are often similar to other common respiratory diseases such as pneumonia. Patients usually seek care from general hospitals rather than the TB dispensary for these symptoms [22]. The integrated model provides TB clinical service in a general hospital, therefore, TB patients seeking care in the hospital can be treated more promptly, and not needed to be referred to the TB dispensaries as per other two models.

Patients in the specialist model had the most complicated patient pathways, reflected by the highest number of health providers visited and the highest medical expenditure. TB specialist hospitals are responsible to treat sophisticated TB cases, such as extra-pulmonary TB, multi-drug resistance cases and cases with complications [20]. All patients in this study were uncomplicated TB cases, and should be treated in the district/county TB dispensary. However, 80% of them were treated in the TB specialist hospitals. One possible reason may be the perceived high quality of the specialist hospital. The specialist hospital may induce more demand as well, because over 70% of hospital revenues came from treating TB patients [23,24]. Therefore, specialists hospitals have the incentive to over-treatment TB patients, and are reluctant to refer patients out.

The logistic regression identified hospitalisation, often unnecessary for uncomplicated TB cases, were the most significant factor associated with longer treatment delays and higher medical expenditure. We found that a significant proportion of patients were hospitalised across the three models. This reflected the public hospitals in China still have the financial incentive to over-treat TB patients despite of the strengthened case reporting system [25]. Internationally, unnecessary and suboptimal TB treatment were also found in large hospitals in Asian and African countries, including unnecessary hospitalisation, not using the DOTS regiment and not referring patients to the national TB programme [26-28]. This needs close attention of the international TB community because improper treatment such as discontinuation of DOTS regiments and early application of second line anti-TB drugs in the hospitals may lead to the emergence or amplification of multi-drug resistance TB [29].

China’s TB control is at a cross-road again after 20 years development through the DOTS program. TB clinical service in the TB dispensary has to be shifted to hospitals, because the Law of Licensing Medical Practitioners was issued, and also because the TB dispensary was generally lack of clinical competences. One argument is to shift all TB treatment to specialist hospitals where available. Our study suggested the potential serious consequences. On the other hand, the study suggesting shift TB clinical service to general hospitals should be a better solution. One worry is that the designated hospitals may over-test and treat patients for profit reasons, as public hospitals may do in the TB dispensary model [22,30]. We found that treatment of TB patients in designated hospitals was in better compliance with national guidelines. This may because of the relatively small number of TB patients compared with of the total patients in the designated hospital. We believe that better government support and regulation enforcement should play a major role in our selected sites. Local government should provide staff salaries and operational costs of the TB clinic in the designated hospital; while the TB dispensary should have a close monitoring and evaluation of case management in the designated hospitals, as outlined in the PPM model [31]. The integrated model has been piloted in poorer western provinces with financial support from the Global Fund. The ceasing of Global Fund support in China in 2013 will pose a threat to the development of integrated model because the local government in poorer provinces may not substitute the essential financial support.

Previous studies in China indicated that financial constraints, rural residence and other socio-demographic factors affected delays in diagnosis and treatment of TB [22,32]. Other policy options may also be explored to improve the accessibility of TB care. Decentralising the TB care from the county level to the community level has demonstrated shortened TB patient care-seeking pathways, reduced patient costs, and improved quality of TB care in China [14,33].

Several limitations have to be borne in mind. Only two sites in each model were selected in the study, great cautions should be taken in extrapolation the results of this study. Substantial variations on income among sites should be taken with caution and may influence results. The two sites in the integrated model were richer than other sites, mainly due to the reason that the majority of established integrated models were only available from more developed provinces or rich areas in the relatively less developed provinces at the time of the study. This may indicate that government support was essential for a functional integrated model. Patient reporting on income, expenditure, and time may suffer from validity problems due to recall bias. The problem was minimised as most interviews were taken within half a year after treatment completion.

## Conclusion

Specialist hospital treating uncomplicated cases not using the standard regimens posed a threat to TB control. The integrated model has shortened patient treatment pathways, and reduced patient costs; therefore, it could be considered as the direction for future reform of China’s TB control system.

## Competing interests

The authors declare that they have no competing interests.

## Authors’ contribution

XW, GZ, JY, JW and QS have designed the research and tools, oversaw the study, and analysed data.XW, JY andGZ have written the manuscript. JW and QS have provided critical comments and revised the manuscript. All authors read and approved the final manuscript.

## Pre-publication history

The pre-publication history for this paper can be accessed here:

http://www.biomedcentral.com/1471-2334/13/93/prepub
